# Choline Fatty Acid Ionic Liquids Enhance Growth, Nitrogen Metabolism, and Grain Guality in Maize (*Zea mays* L.)

**DOI:** 10.3390/molecules31121998

**Published:** 2026-06-07

**Authors:** Qiuchen Guo, Wenquan Chen, Mengfei Niu, Shixu Yang, Yanan Huang, Pei Zhang, Yulong Ma, Qingru Cai, Yajun Li, Xiaohong Chen

**Affiliations:** 1 College of Agronomy, Northwest A & F University, Yangling 712100, China; 2024050026@nwafu.edu.cn (Q.G.); m15167780424@163.com (W.C.); yangshixu@nwafu.edu.cn (S.Y.); huangyanan0801@163.com (Y.H.); pei_zhang1224@163.com (P.Z.); 18234425521@163.com (Y.M.); 15034639628@163.com (Q.C.); 2 College of Life Sciences, Northwest A & F University, Yangling 712100, China; 15931763331@163.com

**Keywords:** ionic liquids, nitrogen metabolism, transcriptomics, crop growth, pentane diacid

## Abstract

Choline-based ionic liquids (ILs) have emerged as promising candidates for application in multifaceted avenues, including electrochemistry, biomaterials, and environmental remediation technologies. However, their regulatory effects on the growth of agricultural plants have rarely been discussed. In this study, 14 choline–fatty acid ILs ([Chl][FA] ILs) containing different FA anions were synthesized, and their effects on the maize growth were investigated. Hydroponic experiments revealed that low concentrations (20 mg/L) of dicarboxylic acid-based [Chl][FA] ILs (e.g., choline pentane diacid [Chl][Pent]) significantly promoted maize root and shoot biomass, whereas higher concentrations inhibited it. Specifically, [Chl][Pent] enhanced chlorophyll content without altering Fv/F_0_, upregulated nitrate reductase (NR) and glutamine synthetase (GS) activities, and stimulated the expression of key nitrogen metabolism (NR and GS) and photosynthetic (Rubisco) genes. Pathway analyses of differentially expressed genes indicated that [Chl][Pent] was associated with the upregulation of nitrogen and glycerophospholipid metabolism. [Chl][Pent] increased the average grain yield by 6.88% over two years compared to CK. Field application of [Chl][Pent] increased grain yield and protein accumulation relative to both control and choline chloride treatments. Overall, these findings demonstrate the potential of dicarboxylic acid-based [Chl][FA] ionic liquids as eco-friendly biostimulants for enhancing crop growth, yield, and quality.

## 1. Introduction

Choline-based ionic liquids ([Chl] ILs) constitute a class of designable functional compounds that have recently demonstrated considerable potential for application in diverse fields such as electrochemistry, biocatalysis, biomedicine, and environmental remediation technologies [1,2,3,4]. However, their physiological impact on plants remains largely unexplored. In biological systems, choline derivatives are ubiquitous and play key roles in critical metabolic processes such as lipid metabolism [5]. Studies have confirmed that crop growth and development can be regulated in multiple ways via exogenous amendment of choline analogs. For instance, choline chloride, a common plant growth regulator, improves crop growth, enhances photosynthesis, and increases yield [6], while its derivative betaine functions as a key osmotic protectant under environmental stresses [7]. From a plant nutritional perspective, quaternary ammonium compounds, such as choline analogs, constitute an important class of organic nitrogen in the soil, serving as potential nitrogen sources for both plants and rhizosphere microorganisms [8]. We hypothesized that [Chl] ILs enhance maize growth by modulating nitrogen metabolism and photosynthetic activity.

Choline–fatty acid ILs ([Chl][FA] ILs) are synthesized by pairing choline cations with various small-molecule FA anions. On a related note, FAs and their derivative anions have recently garnered growing interest in the design of ILs owing to their low production costs, favorable physicochemical properties, structural tunability, high biodegradability, and high environmental compatibility [9,10,11]. In biomedical applications, [Chl][FA] ILs are considerably advantageous for use as surfactants, demonstrating low toxicity and high efficiency [12]. When employed as drug delivery vehicles, they demonstrate both excellent biocompatibility and structural flexibility, facilitating the electrostatic adsorption of nano-drug particles, while FA hydrophobic chains form protective layers to not only prevent nanoparticle aggregation but also enhance membrane interactions for improved transmembrane diffusion [11,13]. Some drug-active [Chl][FA] ILs can serve dual functions as carriers and therapeutic agents [14]. They also exhibit significant potential for application in functional food development, such as co-delivery of flavonoids or as novel naturally derived emulsifiers [15,16].

Despite the well-researched and developed applications mentioned earlier, the growth regulation effects of [Chl][FA] ILs on agricultural crops remain largely unexplored—most existing studies focus primarily on agricultural biomass pretreatment [1,17]. More specifically, in contrast to conventional pretreatment methods (e.g., acid/alkali treatment), [Chl][FA] ILs offer advantages such as reduced chemical pollution, lower energy consumption requirements during production, and higher extraction efficiency [18]. Other studies have examined the effects of these ILs on growth regulation or crop protection. For example, Choline ionic liquid based on 2,4-D exhibits better herbicidal efficacy than its commercial counterpart [19]. In tomato cultivation, the choline derivative choline benzothiadiazole (chol-BTH, synthesized from choline and the resistance inducer BTH) has demonstrated superior performance over BTH alone in inducing the expression of disease resistance genes (and thereby enhancing disease resistance) and activating antioxidant capacity [20]. This dual-action effect mirrors that observed in pharmaceutical applications; nevertheless, it remains underexplored in the context of agriculture. FAs are physiologically active substances abundantly present in nature that regulate critical plant metabolic processes, including energy metabolism and stress response [21,22]. Specifically, certain short-chain dicarboxylic acids participate in the tricarboxylic acid (TCA) cycle, helping to maintain the metabolic balance of reactive oxygen species to promote crop growth and stress tolerance [23].

[Chl][FA] ILs exhibit unique cation–anion synergistic effects, offering novel opportunities for crop regulation. Specifically, in fatty acid-derived [Chl][FA] ILs, variations in anionic carbon chain length and carboxyl group number differentially modulate plant metabolic pathways; however, the underlying mechanisms remain unclear. To address this gap, we synthesized a series of 14 structurally tailored [Chl][FA] ILs and systematically evaluated their growth-regulatory effects in maize (*Zea mays* L.). From this series of synthesized compounds, we selected choline pentane diacid as a representative IL for in-depth analysis of its impacts on seedling growth, physiological responses, gene expression patterns, and grain yield and quality parameters. Overall, this study provides mechanistic insights into the agricultural potential of [Chl][FA] ILs as novel biostimulants for the growth of maize crops.

## 2. Results

### 2.1. Effects of Different [Chl][FA] ILs on Maize Seedling Growth

**Figure 1:**
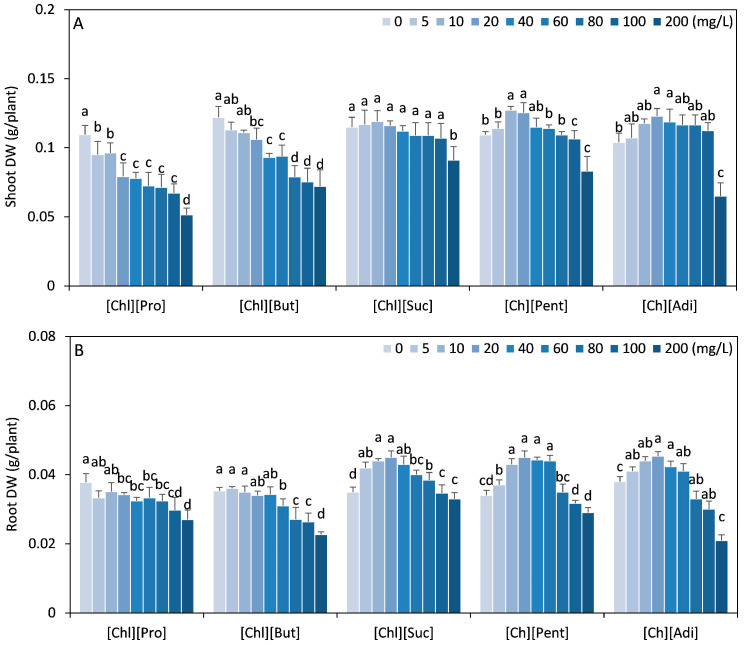
Effects of five [Chl][FA] ILs on maize seedling growth at different concentrations. (**A**,**B**) represent the biomass of the shoots and roots of maize seedlings (a–d represent significant differences across treatments).

A total of 14 [Chl][FA] ILs were synthesized via a one-step neutralization method (as detailed in Table 1). Hydroponically grown maize seedlings were used to evaluate the effects of five representative [Chl][FA] ILs with relative medium alkyl chain lengths ([Chl][Pro], [Chl][But], [Chl][Suc], [Chl][Pent], and [Chl][Adi]) over a concentration gradient of 0–200 mg/L. The results demonstrated that at low concentrations (20 mg/L), three dicarboxylate-based [Chl][FA] ILs ([Chl][Suc], [Chl][Pent], and [Chl][Adi]) promoted seedling growth (measured in terms of root and shoot biomass). However, all five [Chl][FA] ILs exhibited inhibitory effects at higher concentrations (as detailed in Figure 1).

**Figure 2:**
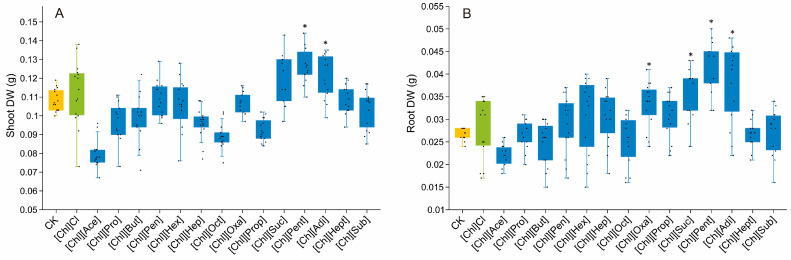
Effects of 14 [Chl][FA] ILs (20 mg/L) on root and shoot dry weight (DW) of maize seedlings. (**A**,**B**) represent the biomass of the shoots and roots of maize seedlings (“*” indicates treatments with values significantly higher than CK at *p* < 0.01).

**Figure 3:**
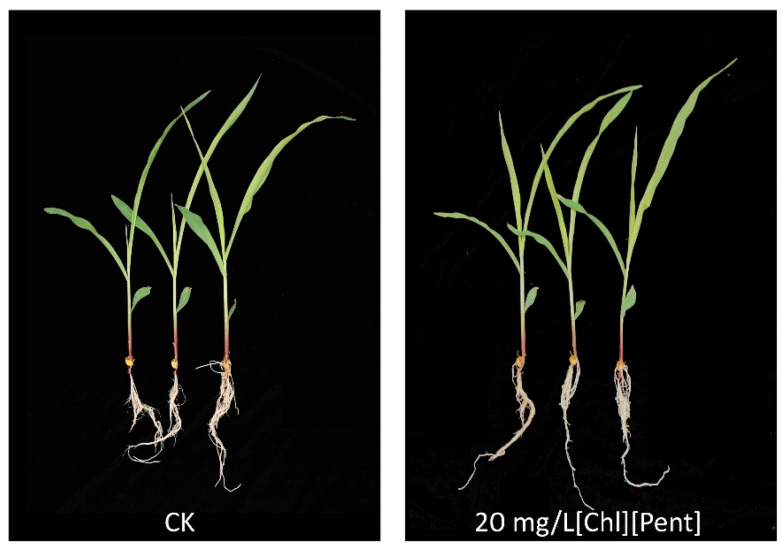
Morphological effects of [Chl][Pent] ILs (20 mg/L) on maize seedling growth.

Based on this, 20 mg/L was selected as the optimal concentration exhibiting maximum growth promotion and subsequently, all 14 synthesized ionic liquids were evaluated at 20 mg/L. The results are presented in Figure 2. [Chl]Cl treatment showed no significant difference from the control (CK) in shoot or root dry weight. Certain [Chl][FA] ILs (e.g., [Chl][Ace], [Chl][Oct]) significantly inhibited maize growth. Some dicarboxylate anion-based ILs demonstrated pronounced growth-promoting effects on maize seedlings, whereas their monocarboxylate counterparts showed no such effects. Specifically, [Chl][Pent] and [Chl][Adi] significantly increased shoot biomass compared to that of CK. Four ILs ([Chl][Oxa], [Chl][Suc], [Chl][Pent], [Chl][Adi]) significantly enhanced root growth, with [Chl][Pent] showing the greatest increase (Figure 2 and Figure 3). Among the 14 examined [Chl][FA] ILs, [Chl][Pent] exhibited the most pronounced growth promotion in maize seedlings, whereas [Chl]Cl alone showed no such activity.

### 2.2. Effects of [Chl][Pent] on Photosynthesis and Nitrogen Metabolism

**Figure 4:**
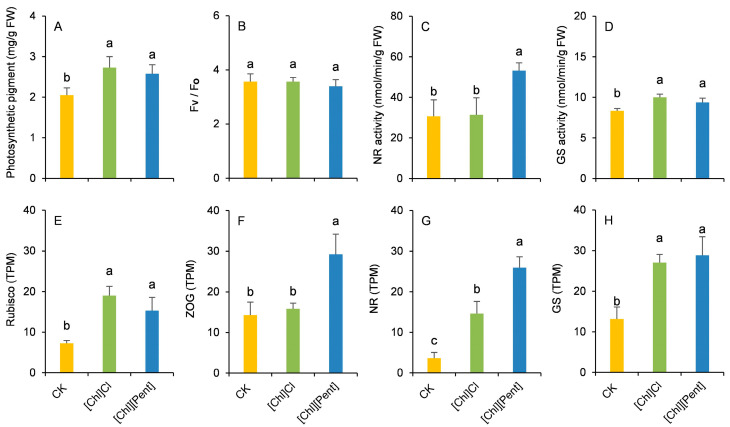
Effects of [Chl][Pent] on leaf photosynthesis pigment contents (**A**), photosynthetic Fv/F_0_ value (**B**), Rubisco gene expression levels (**E**), NR and GS activities (**C**,**D**) and gene expression levels (**G**,**H**), and ZOG gene expression levels (**F**) (NR: nitrate reductase; GS: glutamine synthetase; ZOG: cis-zeatin O-glucosyltransferase; TPM: transcripts per million reads) (a–c represent significant differences across treatments).

The dicarboxylate-based IL [Chl][Pent] was selected for further physiological and mechanistic analyses. Both [Chl]Cl and [Chl][Pent] (at a concentration of 20 mg/L) increased the photosynthetic pigment content but did not alter Fv/F_0_ (Figure 4A,B). Transcript per million (TPM) analyses revealed that photosynthetic Rubisco-encoding genes were upregulated by both [Chl]Cl and [Chl][Pent] treatments (Figure 4E). In the case of nitrogen metabolism, [Chl][Pent] uniquely upregulated NR activity, whereas both [Chl]Cl and [Chl][Pent] treatments enhanced GS activity relative to the CK. Moreover, NR and GS gene expression levels were in agreement with the enzymatic activity data. The gene encoding cis-zeatin O-glucosyltransferase (ZOG) was expressed at significantly higher levels after the [Chl][Pent] treatment than after the CK and [Chl]Cl treatments (as depicted in Figure 4).

### 2.3. Transcriptomic DEGs Analyses and Metabolic Pathway Enrichment

**Figure 5:**
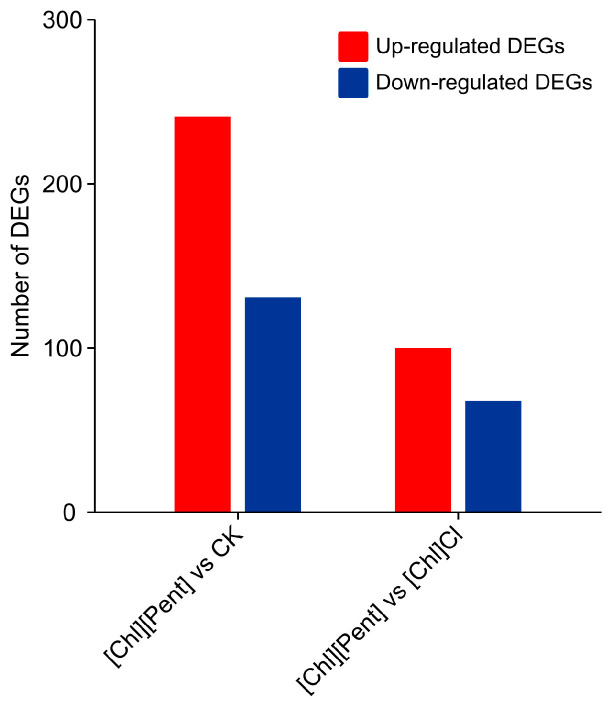
Number of DEGs between [Chl][Pent] and CK, and between [Chl][Pent] and [Chl]Cl.

Comparative transcriptomic analyses were performed to characterize the differential gene expression patterns among the [Chl][Pent] vs. CK and [Chl][Pent] vs. [Chl]Cl treatment scenarios. A total of 345 and 168 DEGs were identified in the [Chl][Pent] vs. CK and [Chl][Pent] vs. [Chl]Cl comparisons, respectively (Figure 5).

The [Chl][Pent] vs. CK scenario comparison is detailed as follows. The 214 upregulated genes were primarily enriched during secondary metabolite biosynthesis, nitrogen metabolism, zeatin biosynthesis, and cutin biosynthesis. The 131 downregulated genes were primarily enriched during phenylpropanoid biosynthesis and secondary metabolite biosynthesis.

The [Chl][Pent] vs. [Chl]Cl scenario comparison is detailed as follows. The 100 upregulated genes were primarily enriched during nitrogen metabolism, secondary metabolite biosynthesis, and the plant mitogen-activated protein kinase signaling pathway. The 68 downregulated genes were enriched during sphingolipid metabolism, linoleic acid metabolism, monoterpenoid biosynthesis, and diterpenoid biosynthesis.

**Figure 6:**
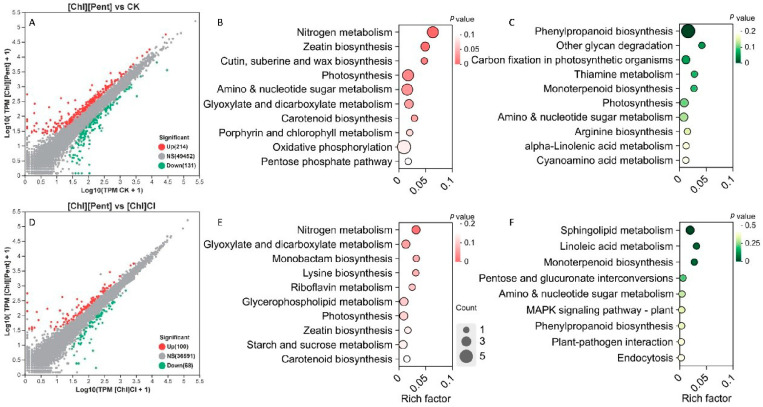
Comparative metabolic pathway enrichment analyses of differentially expressed genes in [Chl][Pent] vs. CK (**A**–**C**) and [Chl][Pent] vs. [Chl]Cl (**D**–**F**) treatments. Panels display significantly enriched metabolic pathways for upregulated (**B**,**E**) and downregulated (**C**,**F**) genes in each comparison.

Furthermore, the observed upregulation of the zeatin biosynthesis and photosynthetic pigment synthesis pathways may contribute to growth enhancement and increased photosynthetic pigment content (as illustrated in Figure 6).

### 2.4. Grain Yield and Quality

The two-year field trials demonstrated that the foliar application of both [Chl][Pent] and [Chl]Cl enhanced maize yield, with [Chl][Pent] treatment yielding superior effects compared to those achieved with [Chl]Cl treatment. The grain quality analyses revealed that [Chl][Pent] increased grain yield in 2024, with effects on grain yield and protein content showing variation across experimental conditions. However, [Chl]Cl had no significant effect on protein content (as detailed in Table 2).

## 3. Discussion

The concentration-dependent experiments revealed that certain [Chl][FA] ILs (e.g., [Chl][Pent]) exhibited a typical “low-promotion, high-inhibition” dose–response trend in hydroponic maize seedlings. This pattern aligns with that for plant hormones and growth regulators, with 20 mg/L identified as the optimal concentration for growth promotion [24]. Among the tested [Chl][FA] ILs, the dicarboxylate-based compounds (e.g., [Chl][Suc] and [Chl][Pent]) consistently demonstrated superior growth-promoting effects compared to those of their monocarboxylate counterparts with equivalent side-chain lengths. This superiority of dicarboxylate anions in plant growth promotion may be ascribed to their structural similarity with that of key TCA cycle intermediates (e.g., succinate and α-ketoglutarate), suggesting potential metabolic modulation [23,25].

Choline, an essential endogenous metabolite in plants, plays critical physiological roles in fatty acid metabolism, membrane stabilization, and stress resistance enhancement [6]. The amphiphilic nature of [Chl][FA] ILs significantly contributes to their plant growth-promoting effects, suggesting that the observed growth benefits may derive from synergistic actions of both choline and fatty acids [24]. Specifically, their high water solubility stimulates growth when they are applied via irrigation or foliar spraying. Additionally, FA moieties are strongly compatible with biological membranes, thereby enabling higher tissue permeability [13]. From a biosafety perspective, studies have demonstrated that choline-based ILs typically display low toxicity and high biodegradability, indicating favorable ecological safety. Moreover, choline compounds in soil are also one of the common organic nitrogen sources [18,26]. In contrast, some common ILs (e.g., imidazolium- and pyridinium-based ILs) have been widely reported to pose significant biotoxicity [27].

The physiological measurements suggest that [Chl][Pent] enhanced photosynthesis and nitrogen metabolism in maize. The increased leaf photosynthetic pigment content was in agreement with the known functions of other choline analogs (e.g., choline chloride, glycine betaine) in stimulating thylakoid development [28,29]. Stability in the Fv/F_0_ ratio (a key indicator of PSII reaction center efficiency) indicates that [Chl][Pent] minimally influenced photochemical efficiency. The result suggests that [Chl][Pent] treatment may promote light-harvesting ability by increasing pigment content and Rubisco activity, rather than regulating PSII efficiency (Fv/F_0_ value). This characteristic clearly distinguishes [Chl][Pent] from toxic agents, such as heavy metals, which typically impair the photosynthetic apparatus.

[Chl][Pent] treatment upregulated key nitrogen metabolism enzymes (NR and GS), potentially through α-ketoglutarate-mediated regulation. As α-ketoglutarate participates in both the TCA cycle and amino acid assimilation pathways, the pentane diacid accumulation induced by [Chl][Pent] may enhance nitrogen metabolism by elevating α-ketoglutarate levels [21,23,30]. Transcriptomic analyses revealed the coordinated upregulation of glycerophospholipid metabolism pathways (Figure 6), which is consistent with the established role of choline as a lipid metabolism intermediate [6,23]. Overall, these findings are consistent with a potential involvement of nitrogen metabolic pathways in the growth-promoting effects of [Chl][Pent].

Field experiments showed that [Chl]Cl and [Chl][Pent] may increase grain yield to some extent, which is consistent with the documented role of choline compounds in promoting photosynthesis and nitrogen metabolism [28,31]. The treatment concentration used in this study is relatively lower and is similar to that of conventional plant growth regulators such as [Chl]Cl, uniconazole and chlormequat chloride [32]. However, from the result of this study, unlike in [Chl]Cl, the presence of the glutarate anion in [Chl][Pent] adds distinct advantages for yield and grain quality improvement. Specifically, the [Chl][Pent] treatment significantly increased the protein content relative to both the control (CK) and [Chl]Cl treatments. This may be correlated with the upregulation of NR-mediated nitrogen assimilation and subsequent protein accumulation, as previously reported [30]. These suggest a potentially dual functional mechanism of [Chl][Pent], as part of which both the choline cation and glutarate anion contribute synergistically to plant growth regulation. For crops such as maize, increased grain protein content typically enhances both nutritional value and marketability. From this perspective, [Chl][Pent] holds distinct advantages over traditional plant growth regulators like [Chl]Cl. In this study, the synthesis procedure for [Chl][Pent] was relatively simple, and its low-concentration application requires minimal dosage, resulting in lower operational costs. These attributes benefit the potential application of [Chl][Pent] as a plant biostimulant in agricultural ecosystems.

## 4. Materials and Methods

### 4.1. Synthesis and Characterization of [Chl][FA] ILs

A total of 14 [Chl][FA] ILs were synthesized via a one-step neutralization reaction using choline hydroxide (Sigma-Aldrich, St. Louis, MO, USA) and various FAs (J&K Scientific Ltd., Guangzhou, China) [33]. Subsequently, the obtained products were characterized using nuclear magnetic resonance (1H NMR) spectroscopy. The synthesized ILs comprised seven monocarboxylic acids and seven dicarboxylic acids with varying side-chain lengths (Table 1). The detailed synthesis procedure followed and NMR spectra visualized are provided in Supplementary File S1.

### 4.2. Hydroponic Maize Growth Experiments

Maize cultivar Zhengdan 958 (*Zea mays* L., purchased from Doneed Seeds Ltd., Beijing, China), a widely employed cultivar in China, was selected for this study. The seeds thereof were surface-sterilized with 75% ethanol and germinated on moist filter paper. Uniformly germinated seeds were transferred to hydroponic boxes (with dimensions of 20 × 13 × 10 cm; eight plants per box) containing 0.5 strength Hoagland nutrient solution (pH 6.0), which was replenished every 48 h.

For preliminary concentration screening, five representative [Chl][FA] ILs were tested at concentrations of 0, 5, 10, 20, 40, 60, 80, 100, and 200 mg/L in the nutrient solution to evaluate their dose-dependent effects on seedling growth. The plants were cultivated for 14 days under controlled conditions (16/8 h light/dark cycles; 28 °C temperature; 8000 lx irradiance). After 14 days, six maize seedlings from each treatment group were taken for root and stem biomass data determination.

Following the preliminary screening, choline pentane diacid ([Chl][Pent]), a representative IL with a dicarboxylate anion, was selected for further investigation. Two controls were included: (1) no additive (CK) and (2) Choline chloride ([Chl]Cl). Fresh leaf samples were collected to evaluate photosynthesis, nitrogen metabolism, and gene expression profiles (the number of biological replicates was all ≥4). The following analytical methods were employed.

Photosynthetic pigments (chlorophylls and carotenoids) were extracted from fresh leaves using 80% acetone and quantified by a three-wavelength spectrophotometric method according to Lichtenthaler (1987) [31]. The chlorophyll fluorescence Fv/F_0_ was measured using an FC 800-C/1010 imaging system (PSI, Drásov, Czech Republic). Nitrate reductase (NR) and glutamine synthetase (GS) activities were determined using commercial assay kits (Comin Biotechnology, Suzhou, China) following the manufacturer’s protocol.

For transcriptomic profiling, total RNA was extracted from the leaves of the CK-, [Chl]Cl-, and [Chl][Pent]-treated plants (at a concentration of 20 mg/L) using the TRIzol reagent. The RNA quality was verified using a NanoDrop 2000 spectrophotometer [34]. Moreover, cDNA libraries were constructed and sequenced using the NovaSeq X Plus platform (Majorbio, Shanghai, China), and gene expression levels were quantified as transcripts per million (TPM). Differentially expressed genes (DEGs) were analyzed using edgeR, and significantly enriched metabolic pathways were identified using KOBAS (http://bioinfo.org/kobas/. accessed on 10 April 2025).

### 4.3. Field Yield and Quality Analyses

A two-year field trial (2023–2024) was conducted during the summer maize growing season (June–October) at the experimental farm of Northwest A&F University, Yangling, Shaanxi, China (34°16′ N, 108°04′ E). Maize was planted at 60,000 plants/ha in a randomized complete block design (*n* = 3, plot size 3 × 7 m^2^). Foliar applications were performed between 09:00–11:00 h under wind speed of 6.5 km/h and temperature of 19–27 °C, with 2023 and 2024 showing comparable weather patterns during application windows (total precipitation of 1164.6 mm in 2023 and 1069.7 mm in 2024; mean temperature of 9–20 °C in 2023 and 10–22 °C in 2024; source of data: https://www.tianqi24.com/yangling (accessed on 29 May 2026). Exogenous choline treatments with CK (water only), [Chl]Cl (100 mg/L), and [Chl][Pent] (100 mg/L) were applied at the early grain-filling stage (10 d after flowering) via foliar spraying. A spraying volume of 450 L/ha was adopted for each treatment. When the crops matured, whole cobs were harvested and air-dried at 25 °C to constant weight, followed by grain yield analysis. Grain quality parameters (i.e., protein, starch, and oil contents) were analyzed using near-infrared spectroscopy (DA7250 platform, Perten, Hägersten, Sweden) [35].

### 4.4. Statistical Analyses

Data are presented as means ± standard deviation (SD) from at least three independent biological replicates. Significant differences among treatments were assessed using one-way ANOVA, followed by Duncan’s multiple range test (*p* < 0.05).

## 5. Conclusions

This study synthesized and investigated the effects of 14 structurally distinct choline fatty acid ionic liquids on hydroponic maize seedling growth. The results showed that low concentration of [Chl][Pent] may promote the growth of maize. Treatment with [Chl][Pent] increased leaf photosynthetic pigment content and nitrogen metabolism enzyme activities. Gene expression profiling revealed that [Chl][Pent] up-regulated genes in growth-associated pathways, such as nitrogen metabolism and zeatin biosynthesis. The field validation results confirmed that foliar application of [Chl][Pent] may improve grain yield and protein content of maize to some extent. These findings show that [Chl][Pent] has translational value as a foliar treatment to enhance maize growth and grain quality under conventional agronomic practices.

## Figures and Tables

**Table 1:** Synthesized 14 [Chl][FA] ILs and their structures.

*Monoacid Anions*			*Diacid Anions*		
*Alkyl Chain*	*Ionic Liquid Name*	*Abbreviation*	*Alkyl Chain*	*Ionic Liquid Name*	*Abbreviation*
C2	Choline acetic acid	[Chl][Ace]	C2	Choline oxalic acid	[Chl][Oxa]
C3	Choline propionic acid	[Chl][Pro]	C3	Choline propane diacid	[Chl][Prop]
C4	Choline butyric acid	[Chl][But]	C4	Choline succinic acid	[Chl][Suc]
C5	Choline pentanoic acid	[Chl][Pen]	C5	Choline pentane diacid	[Chl][Pent]
C6	Choline hexanoic acid	[Chl][Hex]	C6	Choline adipic acid	[Chl][Adi]
C7	Choline heptoic acid	[Chl][Hep]	C7	Choline heptane diacid	[Chl][Hept]
C8	Choline octanoic acid	[Chl][Oct]	C8	Choline suberic acid	[Chl][Sub]

**Table 2:** Effects of [Chl][Pent] on maize yield and quality.

Year	Treatment	Grain Yield (g/Plant)	Starch (%)	Protein (%)	Oleaginousness (%)
2023	CK	123.1 ± 3.2 b	75.00 ± 0.94 a	9.62 ± 0.22 ab	5.02 ± 0.46 a
	[Chl]Cl	128.6 ± 2.8 a	75.85 ± 0.34 a	9.49 ± 0.14 b	5.12 ± 0.16 a
	[Chl][Pent]	131.4 ± 2.3 a	74.83 ± 0.90 a	9.87 ± 0.23 a	4.98 ± 0.50 a
2024	CK	133.8 ± 3.3 c	76.29 ± 0.90 a	8.66 ± 0.26 b	4.52 ± 0.43 a
	[Chl]Cl	138.4 ± 2.1 b	76.45 ± 0.60 a	8.64 ± 0.27 b	4.61 ± 0.24 a
	[Chl][Pent]	143.2 ± 2.4 a	75.58 ± 0.58 a	9.03 ± 0.19 a	4.87 ± 0.13 a

Note: Different letters indicate significant differences within the same year (*p* < 0.05).

## Data Availability

The raw data supporting the conclusions of this article will be made available by the authors on request.

## Supplementary Materials

The following supporting information can be downloaded at https://www.mdpi.com/article/10.3390/molecules31121998/s1, Figure S1: Chemical structures of choline fatty acid ionic liquids [36,37]; Figure S2: The 14 choline fatty acid ionic liquids ([Chl][FA] ILs) synthesized in this study; Table S1: ^1^H NMR data of 14 [Chl][FA] ILs.
