# Supplementary material for: Choline Fatty Acid Ionic Liquids Enhance Growth, Nitrogen Metabolism, and Grain Guality in Maize (Zea mays L.)
Source: Molecules. 2026 Jun 7;31(12):1998. doi: 10.3390/molecules31121998 (PMC13305747; doi:10.3390/molecules31121998)
Supplement: Supplementary file 1 [file molecules-31-01998-s001.zip › molecules-4322531-supplementary.pdf]

Supplementary Information

1. Preparation and characterization of choline fatty acid ionic liquids

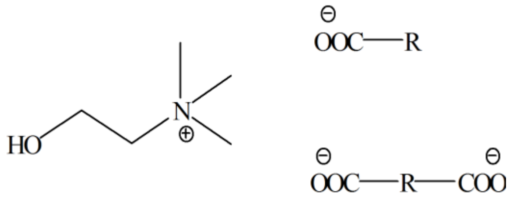

Figure S1. Chemical structures of choline fatty acid ionic liquids.

Choline fatty acid ILs (Figure S1) were synthesized according to previously described methods [1,2] with some modifications. An aqueous solution of choline hydroxide was added drop wise to aqueous solutions of fatty acids in molar ratios of 1：1 (choline hydroxide and monoacids) and 1：2 (choline hydroxide and diacids). The mixtures were stirred for 48 h at 20 °C, followed by evaporation of the water under reduced pressure at 60 °C, and were subsequently stored under moisture-free conditions until utilization. This one-step procedure is a typical atom-economic reaction that does not yield any toxic by-products. All obtained 14 Choline fatty acid ILs were in liquid form at room temperature (Figure S2).

The [Chl][FA] ILs thus obtained were characterized by means of proton nuclear magnetic resonance (^1H NMR) analysis carried out in D_2O using a Bruker AVANCE Digital 500 MHz spectrometer, with tetramethylsilane used as an internal standard. The yields of all the desired products were found to be greater than 95% (Table S1).

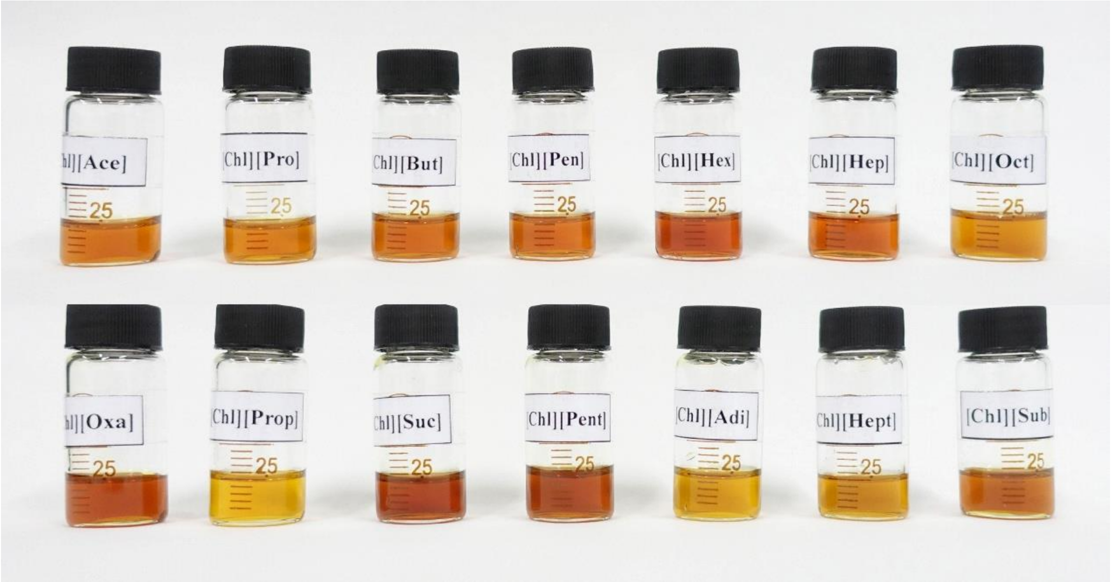

Figure S2. The 14 choline fatty acid ionic liquids ([Chl][FA] ILs) synthesized in this study.

1. [Chl][Ace]: Choline acetic acid
2. [Chl][Pro]: Choline propionic acid
3. [Chl][But]: Choline butyric acid
4. [Chl][Pen]: Choline pentanoic acid
5. [Chl][Hex]: Choline hexanoic acid
6. [Chl][Hep]: Choline heptoic acid
7. [Chl][Oct]: Choline octanoic acid
8. [Chl][Oxa]: Choline oxalic acid
9. [Chl][Prop]: Choline propane diacid
10. [Chl][Suc]: Choline succinic acid
11. [Chl][Pent]: Choline pentane diacid
12. [Chl][Adi]: Choline adipic acid
13. [Chl][Hept]: Choline heptane diacid
14. [Chl][Sub]: Choline suberic acid

2. ^{1}H NMR spectra for seven choline fatty acid ionic liquids

Table S1. ^{1}H NMR data of 14 [Chl][FA] ILs.

ILs	δ/ppm
**[Chl][Ace]**	1.85 (s, 3H, C*H*$_3$), 3.13 (s, 9H, (C*H*$_3$)$_3$), 3.45 (m, 2H, C*H*$_2$), 3.99 (m, 2H, C*H*$_2$).
**[Chl][Pro]**	0.99 (t, *J* = 7.52 Hz, 3H, C*H*$_3$), 2.12 (q, *J* = 7.46 Hz, 2H, C*H*$_2$), 3.14 (s, 9H, (C*H*$_3$)$_3$), 3.45 (s, 2H, C*H*$_2$), 4.00 (s, 2H, C*H*$_2$).
**[Chl][But]**	0.83 (t, *J* = 7.25 Hz, 3H, C*H*$_3$), 1.50 (td, *J* = 14.52, 7.07 Hz, 2H, C*H*$_2$), 2.10 (t, *J* = 7.14 Hz, 2H, C*H*$_2$), 3.13 (s, 9H, (C*H*$_3$)$_3$), 3.45 (s, 2H, C*H*$_2$), 3.99 (s, 2H, C*H*$_2$).
**[Chl][Pen]**	0.83 (t, *J* = 7.24 Hz, 3H, C*H*$_3$), 1.25 (td, *J* = 14.53, 7.30 Hz, 2H, C*H*$_2$), 1.51-1.42 (m, 2H, C*H*$_2$), 2.12 (t, *J* = 7.35 Hz, 2H, C*H*$_2$), 3.14 (s, 9H, (C*H*$_3$)$_3$), 3.46 (d, *J* = 3.52 Hz, 2H, C*H*$_2$), 4.00 (s, 2H, C*H*$_2$).
**[Chl][Hex]**	0.82 (t, *J* = 6.41 Hz, 3H, C*H*$_3$), 1.25 (m, 4H, C*H*$_2$C*H*$_2$), 1.50 (dd, *J* = 14.10, 7.00 Hz, 2H, C*H*$_2$), 2.11 (t, *J* = 7.29 Hz, 2H, C*H*$_2$), 3.15 (s, 9H, (C*H*$_3$)$_3$), 3.46 (s, 2H, C*H*$_2$), 4.00 (s, 2H, C*H*$_2$).
**[Chl][Hep]**	0.84-0.77 (m, 3H, C*H*$_3$), 1.24 (d, *J* = 14.52 Hz, 6H, C*H*$_2$, C*H*$_2$, C*H*$_2$), 1.47 (dd, *J* = 14.46, 7.15 Hz, 2H, C*H*$_2$), 2.10 (q, *J* = 7.71 Hz, 2H, C*H*$_2$), 3.14 (s, 9H, (C*H*$_3$)$_3$), 3.48-3.43 (m, 2H, C*H*$_2$), 4.02-3.97 (m, 2H, C*H*$_2$).
**[Chl][Oct]**	0.81 (t, *J* = 6.73 Hz, 3H, C*H*$_3$), 1.23 (d, *J* = 4.78 Hz, 8H, C*H*$_2$, C*H*$_2$, C*H*$_2$, C*H*$_2$), 1.52-1.45 (m, 2H, C*H*$_2$), 2.11 (t, *J* = 7.49 Hz, 2H, C*H*$_2$), 3.13 (d, *J* = 15.63 Hz, 9H, (C*H*$_3$)$_3$), 3.48-3.44 (m, 2H, C*H*$_2$), 4.00 (m 2H, C*H*$_2$).
**[Chl][Oxa]**	3.14 (s, 18H, (C*H*$_3$)$_3$, (C*H*$_3$)$_3$), 3.49-3.40 (m, 4H, C*H*$_2$, C*H*$_2$), 4.00 (m, 4H, C*H*$_2$, C*H*$_2$).
**[Chl][Prop]**	1.95 (m , 2H, C*H*$_2$), 3.14 (s, 18H, (C*H*$_3$)$_3$, (C*H*$_3$)$_3$), 3.40-3.51 (m, 4H, C*H*$_2$, C*H*$_2$), 4.00 (m, 4H, C*H*$_2$, C*H*$_2$)
**[Chl][Suc]**	2.33 (s, 4H, C*H*$_2$, C*H*$_2$), 3.13 (s, 18H, (C*H*$_3$)$_3$, (C*H*$_3$)$_3$), 3.48-3.40 (m, 4H, C*H*$_2$, C*H*$_2$), 3.99 (m, 4H, C*H*$_2$, C*H*$_2$).
**[Ch][Pent]**	1.74 (dd, *J* = 15.11, 7.57 Hz, 2H, C*H*$_2$), 2.13 (t, *J* = 7.41 Hz, 4H, C*H*$_2$, C*H*$_2$), 3.15 (s, 18H, (C*H*$_3$)$_3$, (C*H*$_3$)$_3$), 3.46 (m, 4H, C*H*$_2$, C*H*$_2$), 4.00 (m, 4H, C*H*$_2$, C*H*$_2$).
**[Ch][Adi]**	1.51-1.45 (m, 4H, C*H*$_2$, C*H*$_2$), 2.12 (t, *J* = 6.66 Hz, 4H, C*H*$_2$, C*H*$_2$), 3.13 (s, 18H, (C*H*$_3$)$_3$, (C*H*$_3$)$_3$), 3.47-3.42 (m, 4H, C*H*$_2$, C*H*$_2$), 4.03-3.94 (m, 4H, C*H*$_2$, C*H*$_2$).
**[Ch][Hept]**	1.53-1.45 (m, 4H, C*H*$_2$, C*H*$_2$), 2.11 (t, *J* = 7.53 Hz, 4H, C*H*$_2$, C*H*$_2$), 3.13 (s, 18H, (C*H*$_3$)$_3$, (C*H*$_3$)$_3$), 3.48-3.42 (m, 4H, C*H*$_2$, C*H*$_2$), 3.99 (d, *J* = 4.39 Hz, 4H, C*H*$_2$, C*H*$_2$).
**[Ch][Sub]**	1.25 (s, 4H, C*H*$_2$, C*H*$_2$), 1.49 (s, 4H, C*H*$_2$, C*H*$_2$), 2.11 (t, *J* = 7.15 Hz, 4H, C*H*$_2$, C*H*$_2$), 3.14 (s, 18H, (C*H*$_3$)$_3$, (C*H*$_3$)$_3$), 3.45 (s, 4H, C*H*$_2$, C*H*$_2$), 3.99 (s, 4H, C*H*$_2$, C*H*$_2$).

References

1. Liu, Q.P.; Hou, X.D.; Li, N.; Zong, M.H. Ionic liquids from renewable biomaterials: synthesis, characterization and application in the pretreatment of biomass. *Green Chem.* **2012,** *14*, 304–307.
2. Moriel, P.; Garcia-Suarez, E.J.; Martinez, M.; Garcia, A.B.; Montes-Moran, M.A.; Calvino-Casilda, V.; Banares, M.A. Synthesis, characterization, and catalytic activity of ionic liquids based on biosources. *Tetrahedron Lett.* **2010**, *51*, 4877–4881.
